# Prolonged Changes in Hepatic Mitochondrial Activity and Insulin Sensitivity by High Fructose Intake in Adolescent Rats

**DOI:** 10.3390/nu13041370

**Published:** 2021-04-19

**Authors:** Arianna Mazzoli, Cristina Gatto, Raffaella Crescenzo, Luisa Cigliano, Susanna Iossa

**Affiliations:** Department of Biology, University of Naples Federico II, Complesso Universitario Monte Sant’Angelo, 80126 Naples, Italy; arianna.mazzoli@unina.it (A.M.); cri.gatto51@gmail.com (C.G.); rcrescen@unina.it (R.C.); luisa.cigliano@unina.it (L.C.)

**Keywords:** hepatic mitochondria, SCD-1, insulin resistance, energy balance, body lipids

## Abstract

Persistence of damage induced by unhealthy diets during youth has been little addressed. Therefore, we investigated the impact of a short-term fructose-rich diet on liver metabolic activity in adolescent rats and the putative persistence of alterations after removing fructose from the diet. Adolescent rats were fed a fructose-rich diet for three weeks and then switched to a control diet for further three weeks. Body composition and energy balance were not affected by fructose-rich diet, while increased body lipids and lipid gain were found after the rescue period. Switching to a control diet reversed the upregulation of plasma fructose, uric acid, lipocalin, and haptoglobin, while plasma triglycerides, alanine aminotransferase, lipopolysaccharide, and tumor necrosis factor alpha remained higher. Hepatic steatosis and ceramide were increased by fructose-rich diet, but reversed by returning to a control diet, while altered hepatic response to insulin persisted. Liver fatty acid synthase and stearoyl-CoA desaturase (SCD) activities were upregulated by fructose-rich diet, and SCD activity remained higher after returning to the control diet. Fructose-induced upregulation of complex II-driven mitochondrial respiration, peroxisome proliferator-activated receptor-gamma coactivator 1 alpha, and peroxisome proliferator activated receptor α also persisted after switching to control diet. In conclusion, our results show prolonged fructose-induced dysregulation of liver metabolic activity.

## 1. Introduction

Fructose, besides being found in many fruits, is often used as a sweetener, mainly in the form of high-fructose corn syrup (HFCS) (whose fructose content is mainly 55% (in HFCS-55). This compound, due to the low cost, became widely used for the preparation of soft drinks, baking products, and ice creams [1], thus contributing to the worldwide increase in fructose intake [2]. Interestingly, this increase coincides with the obesity epidemic [3]. More concerning is the increasing consumption of fructose in the young population. Intakes of added sugars, in the form of soft drinks, fruit-based drinks, and sweet bakery products ranges from less than 3% to 18% in children 4–10 years, while in adolescents it ranges from 13.6% to 16.6% [4]. Increased fructose intake is associated not only with increased obesity risk, but also with fat deposition in the liver and insulin resistance [5,6]. This worrying trend has been also observed in Italy, where the majority (83%) of children consume quantities of simple carbohydrates above the limits of the Italian Dietary Reference Values [7]. Given this situation, understanding whether fructose deleterious consequences are confined to the periods of high intake or persist after switching to a control diet is of outmost importance, especially for the young populations.

It has long far known that the liver is the main tissue target of increased fructose intake [8]. Very recently, it has been demonstrated that fructose reaches the liver when its dietary intake exceeds the intestinal clearance capacity, with the resulting fructose spillover driving metabolic syndrome [9,10]. Using an animal model of increased fructose intake, we have previously shown that long-term feeding with a fructose-rich diet in adult rats elicits obesity (i.e., increased body fat mass) and insulin resistance, together with increased de novo lipogenesis, altered mitochondrial function, and oxidative stress in the liver [11,12,13]. More recently, we have found that similar metabolic alterations can be detected after shorter periods of high fructose intake in adult rats, while young rats display metabolic derangements in the liver without developing obesity [14]. 

Taking into account the above considerations, we delineated the impact of a short-term (three weeks) fructose-rich diet on liver metabolic activity, with a special focus on lipid handling and mitochondrial function, in adolescent rats. In addition, a major aim of this study was to elucidate whether the observed hepatic alterations are prolonged or reversible after removing fructose from the diet. To this end, 30 days-old rats were fed a fructose-rich diet for three weeks and then switched to a control diet for further three weeks. After the two dietary periods, metabolic profiling was obtained by assessing body composition, energy balance and macronutrient partitioning, together with plasma metabolic and inflammatory markers. Liver physiology was evaluated by determining tissue composition, insulin sensitivity, the activity of de novo lipogenesis enzymes, mitochondrial respiratory capacity and protein regulators of hepatic metabolism, namely peroxisome proliferator-activated receptor-gamma coactivator 1 alpha (PGC1α) and peroxisome proliferator activated receptor alpha (PPARα).

## 2. Materials and Methods

### 2.1. Animals and Treatments

**Figure 1:**
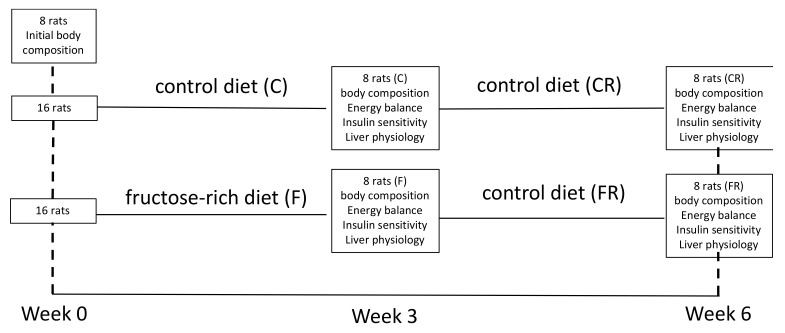
Experimental design.

Male Wistar rats (Charles River, Italy), of 30 days of age were maintained in a temperature-controlled room (23 ± 1 °C) with a 12-h light/dark cycle (06:30–18:30). At the beginning of the experiments, all rats were weighed and then divided in three groups (P0 *n* = 8, C *n* = 16, F *n* = 16) exhibiting the same mean body weight. The P0 group was euthanized at the beginning of the experimental period to measure initial body composition. C group was fed a control diet for three weeks and F group was fed a fructose-rich diet for three weeks. At the end of this period, C rats were weighed and divided in two subgroups, each exhibiting the same mean body weight, and the same was done with F rats. One subgroup of C and F rats was euthanized to analyze the effect of the fructose-rich diet, while the other subgroup of C and F rats was fed a control diet for further three weeks, and called CR and FR rats, respectively. The composition of the two diets is shown in Table 1, and a scheme of the experimental design is shown in Figure 1. During the treatment, body weight and food intake were monitored daily. At the end of the experimental period, the rats were anesthetized with sodium pentothal (40 mg kg^−1^ i.p.), euthanized by decapitation and livers were immediately collected. Liver samples were snapfrozen and stored for further analyses or freshly processed for mitochondrial assays or used to prepare paraffin embedded sections, as described below. 

### 2.2. Glucose Tolerance Test

Blood samples were collected from a small tail clip in EDTA-coated tubes, both after 6 h of fasting and 15 and 30 min after intraperitoneal glucose injection (2 g kg^−1^). Blood samples were centrifuged at 1400× *g* for 8 min at 4 °C, plasma was collected and stored at −20 °C for further determination of glucose and insulin. Plasma glucose concentration was measured by a colorimetric enzymatic method (GS Diagnostics SRL, Guidonia Montecelio, Rome, Italy). Plasma insulin concentration was measured using an ELISA kit (Mercodia AB, Uppsala, Sweden). We measured the area under the curve (AUC) of plasma glucose and insulin during the period 0–30 min of the glucose load. Then, hepatic insulin resistance index was calculated as = (glucose AUC 0–30) × (insulin AUC 0–30) [15].

### 2.3. Body Composition and Energy Balance

Body composition was assessed as previously reported [16]. Briefly, the gut was cleaned of undigested food and the carcasses were then autoclaved, diluted in distilled water and homogenized. Energy content was measured in samples of the homogenized carcasses by bomb calorimetry. Body lipid content was measured by the Folch method [17]. Body protein content was determined using a formula relating total energy value of the carcass, energy derived from fat, and energy derived from protein, using the caloric values of 39.2 kJ/g and 23.5 kJ/g, for body fat and protein, respectively [18]. Metabolizable energy (ME) intake was determined by subtracting the energy measured in feces and urine from the gross energy intake, determined from daily food consumption and gross energy density of the diet. Body energy, fat and protein gain were calculated as the difference between the final and initial content of body energy, fat, and protein. Energy expenditure was determined as the difference between ME intake and energy gain, and energetic efficiency was calculated as the percentage of body energy retained per ME intake. Lipid and protein partitioning was obtained by calculating the amount (expressed in %) of lipid or protein intake that was stored or oxidized.

### 2.4. Metabolic Parameters in Plasma and Liver

Blood samples collected at the time of euthanasia in EDTA-coated tubes were centrifuged at 1400× *g* for 8 min at 4 °C, plasma was isolated and stored at −20 °C. Colorimetric enzymatic methods were used to assess metabolites by using commercial kits (Sigma Aldrich, St. Louis, MO, USA for fructose, GS Diagnostics SRL, Guidonia Montecelio, Rome, Italy for uric acid, SGM Italia, Rome, Italy for alanine aminotransferase (ALT) and triglycerides).

Plasma lipopolysaccharide (LPS) levels were determined using a kit based on a limuls amaebocyte lysate (LAL) extract (ThermoFisher Scientific, Rockford, IL, USA). In brief, samples were incubated with the LAL reagent for 10 min at 37 °C. Then, the chromogenic substrate solution was added for 6 min at 37 °C, reaction was stopped with stop reagent and the absorbance of the samples was assessed on a plate reader at 405 nm.

Plasma tumor necrosis factor alpha (TNFα) concentrations were determined by enzyme linked immunosorbent assay (ELISA) (R&D Systems, Minneapolis, MN, USA) according to manufacturer’s instruction.

Liver triglycerides were assessed as described above for plasma samples in hepatic homogenates prepared in KCl 175 mM, Tris 10 mM, pH 7.5 (1:8 *w/v*) by using Potter Elvehjem homogenizer set at 800 rpm (4 strokes in 1 min). 

Hepatic steatosis was evaluated by hematoxylin and eosin staining of paraffin embedded sections. For the analysis, images were acquired with 20× magnification, 3 random field/section per rat were analysed and scored blindly. 

Liver ceramide content was quantified by ELISA as previously described [19]. Briefly, hepatic lipids extracted with the Folch method were adsorbed to well bottoms of an ELISA microplate overnight at 4 °C. Plates were blocked with 10 mM PBS, 140 mM NaCl, 0.1% Tween, pH 7.4 supplemented with 1% bovine serum albumin for 1 h at 37 °C. The plates were then washed three times with 10 mM PBS, 140 mM NaCl, 0.05% Tween, pH 7.4 (Tween-PBS) and incubated with monoclonal anti-ceramide antibody (Sigma, MO, USA, 2 μg/mL) for 1 h at 37 °C. After three washings in Tween-PBS, peroxidase-conjugated goat anti-mouse IgM (Sigma, MO, USA, 1:5000 dilution) was incubated for 1 h at 37 °C. After four washings in Tween-PBS, the wells were incubated with 100 μl of a color development solution (20 mg of o-Phenylenediamine dihydrochloride in 50 mL of 70 mM Na_2_HPO_4_, 30 mM citric acid, pH 5, supplemented with 120 μl of 3% H_2_O_2_), for 15 min at 37 °C. The reaction was then stopped with 50 μl of 2.5 M H_2_SO_4_ and the absorbance was measured at 492 nm. All tests were done in triplicate. Immunoreactivity was normalized to starting tissue weight. Negative control reactions included omission of primary antibody.

### 2.5. Hepatic Mitochondrial Function 

Liver samples were homogenized (1:1000, *w/v*) in Mir05 medium containing 110 mM sucrose, 60 mM K-lactobionate, 20 mM Hepes, 20 mM taurine, 10 mM KH_2_PO_4_, 6 mM MgCl_2_, 0.5 mM EGTA, 0.1% fatty acid free BSA, pH 7.0 by using Potter Elvehjem homogenizer set at 800 rpm (four strokes in 1 min).

A Substrate, Uncoupler, Inhibitor Titration (SUIT) protocol was used to evaluate mitochondrial function on liver homogenates, by using O_2_k (Oroboros Instruments, Innsbruck, Austria) Measurements were carried out at 37 °C. 

Firstly, leak respiration involving complex I-linked electron flow was assessed by adding malate (0.5 mM), pyruvate (5 mM), and glutamate (10 mM). Phosphorylating respiration with complex I-linked substrates was measured after the addition of ADP 2.5 mM. Succinate 10 mM was then added to measure phosphorylating respiration with electron input from complex I and II, while maximum capacity of the electron transport chain was assessed by adding oligomycin at 2.5 µM (to inhibit ATP synthase), followed by the uncoupler carbonylcyanide p-trifluoromethoxyphenyl-hydrazone (FCCP, 0.5 µM). Maximal capacity supported by complex II alone was calculated after addition of Rotenone (0.5 µM) to inhibit complex I. Correction for non-mitochondrial respiration was carried out by subtracting residual oxygen consumption obtained after the addition of the inhibitor Antimycin A (2.5 µM). Stimulation of complex I-linked respiration by 10 mM exogenous cytochrome c was tested to evaluate mitochondrial integrity.

### 2.6. Hepatic Lipogenesis Enzymes

Liver samples, homogenised in KCl 175 mM, Tris 10 mM, pH 7.5 (1:8 *w/v*) by using Potter Elvehjem homogenizer set at 800 rpm (four strokes in 1 min), were used to assess the enzymatic activity of Stearoyl CoA desaturase (SCD) and Fatty acid synthase (FAS). 

SCD activity was measured palaeographically at 37 °C in a medium containing 0.1 M K_2_HPO_4_, pH 7.4, 1 μM myxothiazol, 0.12 mM NADH and 0.06 mM stearoyl CoA. Oxygen consumption was measured in the presence of myxothiazol, an inhibitor of mitochondrial respiration (to avoid mitochondrial contribution to oxygen consumption) both in the absence and in the presence of cyanide 5 mM, and SCD activity was calculated as the difference between the values in the absence and in the presence of cyanide, since it is known that oxygen consumption by SCD is cyanide-sensitive [20]. 

FAS activity was measured according to Penicaud et al. [21]. Briefly, absorbance at 340 nm was measured in samples incubated in the presence of KH_2_PO_4_ 0.1 M, pH 6.5, acetyl-CoA 60 μM, malonil-CoA 90 μM, and NADPH 300 μM. One unit of FAS activity was defined as that degrading 1 μmol of NADPH per minute at 37 °C.

### 2.7. Western Blotting of Plasma and Hepatic Proteins

Liver tissue samples were diluted 1:1 with lysis buffer (20.0 mM Tris, pH 8, 5% glycerol, 138 mM NaCl, 2.7 mM KCl, 1% NP-40, 5 mM EDTA, 5% protease inhibitor cocktail, 1% phosphatase inhibitor cocktail), and then centrifuged at 15,000× *g* for 15 min at 4 °C. Supernatants were then collected and used for the quantification of several proteins, namely haptoglobin, lipocalin, p-Akt, Peroxisome proliferator-activated receptor-gamma coactivator 1 alpha (PGC1α), peroxisome proliferator activated receptor alpha (PPARα). 

After the determination of protein concentration by the method of Hartree [22], samples of plasma (40 µg) or protein extracts from livers (20 µg) were denaturated in Laemmli’s buffer (60 mM Tris pH 6.8, 10% sucrose, 2% SDS, 4% β-mercaptoethanol, 0.02% bromophenol blue) and loaded on a 10% SDS–polyacrylamide gel. After the run, the gels were transferred on polyvinylidene difluoride membranes (Millipore, Billerica, MA, USA) at 0.5 mA/cm2 for 120 min. The membranes were preblocked in PBS, 3% bovine albumin serum, 0,3% Tween 20 for 1 h and then incubated overnight at 4 °C with antibodies for p-Akt (Cell Signaling, Danvers, MA, USA, code n. 4060; diluted 1:1000 in blocking buffer), lipocalin (Thermo Fisher, Rock Ford, USA, code n. PA5-46938; diluted 1:200 in blocking buffer), haptoglobin (Sigma-Aldrich, ST. Louis, MO, USA, code no. AV42218; diluted 1:500 in blocking buffer), PGC1α (Millipore, Billerica, MA, USA, code no. AB3242; 1:1000 in blocking buffer) or PPARα (Thermo Fisher, IL, USA, code n. PA1-32484; 0,5 mg/mL in blocking buffer). Membranes were washed and then incubated for 1 h at room temperature with secondary antibodies (Promega, Madison, WI, USA, code no. W4021 (anti-rabbit) or V8051 (anti-goat) diluted 1:5000). The membranes were then washed and incubated at room temperature with a chemiluminescent substrate, Immobilon (Millipore Corporation, Billerica, MA, USA). For loading control, Akt was detected with polyclonal antibody (Cell Signaling, Danvers, MA, USA, code no. 9272S; diluted 1:1000 in blocking buffer) and used to normalize the p-Akt signal, actin was detected with polyclonal antibody (Sigma-Aldrich, St Louis, MO, USA, code n. A2066; diluted 1:1000 in blocking buffer) and used to normalize the PGC1α and PPARα signals. Quantitative densitometry of the bands was carried out by analyzing chemidoc images using Image Lab Software (Biorad Laboratories S.r.l., Segrate, Italy). The data of each marker are normalized to controls. 

### 2.8. Statistical Analysis

Data are reported as mean values ± SEM. The program GraphPad Prism 6 (GraphPad Software, San Diego, CA, USA) was used to verify that raw data have normal distribution and to perform one-way ANOVA followed by Tukey post-test or linear regression analysis. A probability < 5% (*p* < 0.05) was considered statistically significant in all analyses.

## 3. Results

### 3.1. Body Composition and Energy Balance

**Figure 2:**
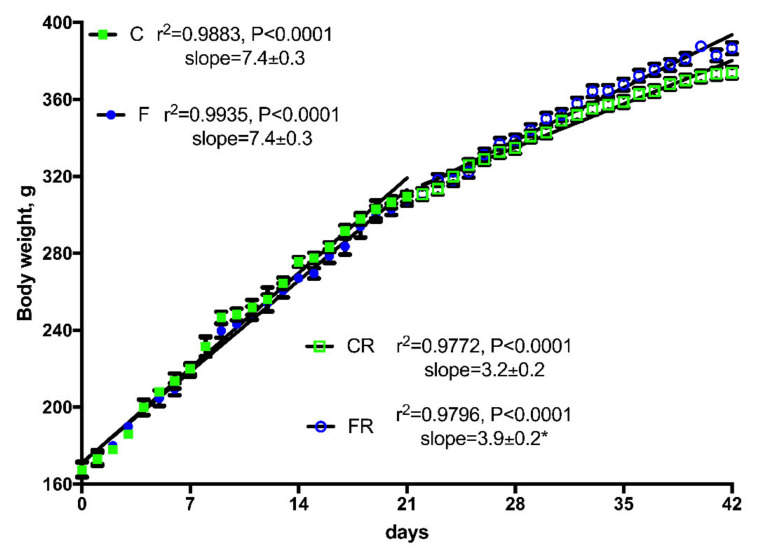
Body weight gain in control (C), fructose (F), control rescue (CR), and fructose rescue (FR) rats. Values are the means ± SEM of 16 (C and F) or eight (CR and FR) different rats. Linear regression analysis showed that line for FR rats was significantly different (*p* < 0.05) from line for CR rats. * Slope from FR rats was significantly higher (*p* < 0.05) than that from CR rats.

Body composition determination revealed that after three weeks of fructose-rich diet no changes were evident in final body weight, body weight gain and energy gain between C and F rats (Table 2). Surprisingly, when fructose-fed rats were returned to control diet consumption for additional three weeks, final body weight and the amount of epididymal and retroperitoneal fat were increased in FR rats compared to CR rats (Table 2). In addition, during the rescue period, both CR and FR rats decreased their body weight gain rate, although the slope of the line (indicative of daily weight gain) was significantly higher in FR rats compared to CR rats (Figure 2). This difference in weight gain rates between CR and FR rats might be due to the subtle decrease in energy expenditure (although not significant) found in FR rats and reported in Table 2.

To get further insight into the changes in body composition, we analyzed the main components of energy balance (Table 2). All the measured parameters of energy balance were not affected by three weeks of fructose-rich diet, as we found no difference between C and F rats, while after the rescue period a significant increase in body energy gain was found in FR rats (Table 2). 

**Figure 3:**
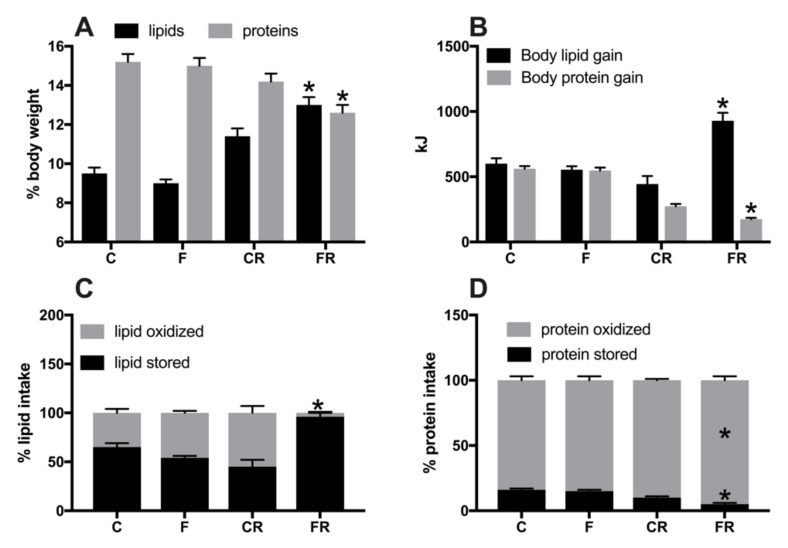
Body lipid and protein content (**A**), body lipid and protein gain (**B**), lipid (**C**) and protein (**D**) partitioning in control (C), fructose (F), control rescue (CR), and fructose rescue (FR) rats. Values are the means ± SEM of eight different rats. * *p* < 0.05 compared to respective control (one-way ANOVA followed by Tukey post-test).

Parameters of body composition, namely body lipids and proteins (Figure 3A) were significantly higher in FR rats, that also exhibited significantly higher values of lipid gain (Figure 3B), while protein gain was significantly lower (Figure 3B). From macronutrient partitioning data, it emerged that the increased body lipid deposition found in FR rats arose from lower lipid oxidation in favor of lipid gain (Figure 3C), coupled with a decreased protein deposition in favor of protein oxidation (Figure 3D). 

### 3.2. Plasma Metabolic and Inflammatory Profile

**Figure 4:**
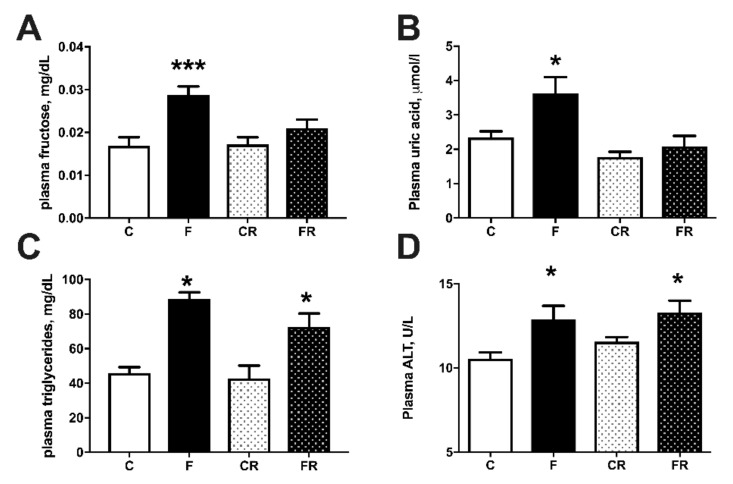
Plasma metabolic parameters: fructose (**A**), uric acid (**B**), triglycerides (**C**), and alanine aminotransferase (ALT) (**D**), in control (C), fructose (F), control rescue (CR) and fructose rescue (FR) rats. Values are the means ± SEM of eight different rats. * *p* < 0.05, *** *p* < 0.001 compared to respective control (one-way ANOVA followed by Tukey post-test).

Metabolic characterization of the four groups of rats was obtained by measuring several plasma markers (Figure 4). Plasma concentrations of fructose (Figure 4A) and uric acid (Figure 4B) significantly increased in F rats, but returned to control levels in FR rats, while plasma levels of triglycerides (Figure 4C) were increased by fructose-rich diet in F rats and remained higher even after the cessation of this diet in FR rats, compared to CR rats. Plasma levels of ALT (Figure 4D) were assessed as marker of liver damage [23] and found significantly higher both at the end of high fructose feeding in F rats and three weeks after returning to control diet in FR rats.

**Figure 5:**
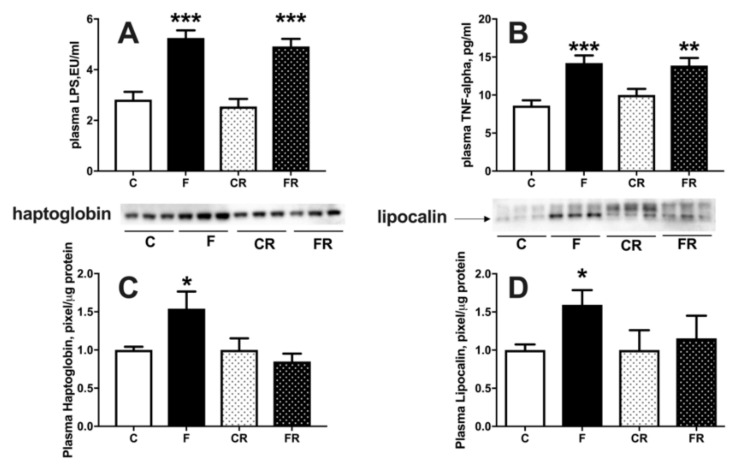
Plasma markers of inflammation: lipopolysaccharide (LPS) (**A**), tumor necrosis factor alpha (TNF-α) (**B**), haptoglobin (**C**), with representative western blot, normalized to control) and lipocalin (**D**), with representative western blot, normalized to control) in control (C), fructose (F), control rescue (CR) and fructose rescue (FR) rats. Values are the means ± SEM of eight different rats. * *p* < 0.05, ** *p* < 0.01, *** *p* < 0.001 compared to respective control (one-way ANOVA followed by Tukey post-test).

Metabolic derangement is usually associated with systemic inflammation [24] and therefore we measured LPS, TNFα, lipocalin, and haptoglobin as markers of inflammation (Figure 5). Plasma levels of LPS (Figure 5A) and TNFα (Figure 5B) were significantly increased in F rats, and remained significantly higher also in FR rats, while haptoglobin (Figure 5C) and lipocalin (Figure 5D) were upregulated in F rats but returned to control levels in FR rats.

### 3.3. Hepatic Insulin Sensitivity and De Novo Lipogenesis

**Figure 6:**
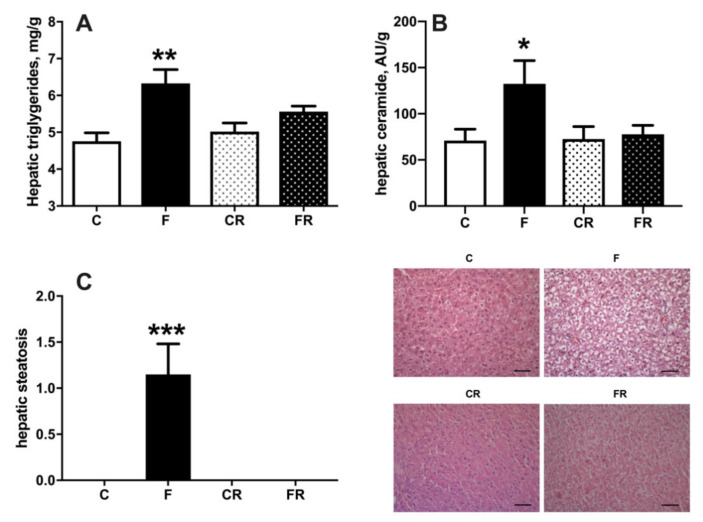
Triglycerides (**A**), ceramide (**B**), and degree of steatosis (**C**), in liver from control (C), fructose (F), control rescue (CR), and fructose rescue (FR) rats. Values are the means ± SEM of eight different rats. * *p* < 0.05, ** *p* < 0.01, *** *p* < 0.001 compared to respective control (one-way ANOVA followed by Tukey post-test). Scalebar = 50 μm.

Increased fructose intake mainly impacts on liver function, due to its ability to metabolize this substrate [25]. We therefore evaluated liver composition, together with primary aspects of its functionality, namely sensitivity to the hormonal action of insulin and capacity of de novo lipogenesis. Assessment of hepatic composition revealed that after three weeks of fructose-rich diet the content of triglycerides (Figure 6A), ceramide (Figure 6B), and steatosis (Figure 6C), were all significantly increased in F rats, while all these modifications were completely reversed after switching to a control diet in FR rats.

**Figure 7:**
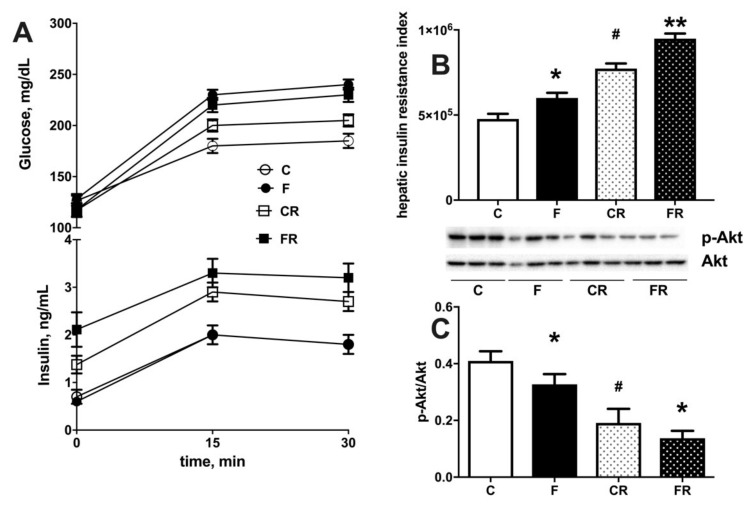
Plasma glucose and insulin during 30 min after intraperitoneal injection of glucose (**A**), hepatic insulin resistance index (**B**) and degree of phosphorylation of kinase Akt (with representative western blot) (**C**) in liver from control (C), fructose (F), control rescue (CR) and fructose rescue (FR) rats. Values are the means ± SEM of eight different rats. * *p* < 0.05, ** *p* < 0.01 compared to respective control; # *p* < 0.05 compared to C rats (one-way ANOVA followed by Tukey post-test).

Both increased cellular triglycerides and ceramide are considered key players in establishing insulin resistance [26]. We therefore injected glucose intraperitoneally and established time course of plasma glucose and insulin in the following 30 min. During this initial period after glucose injection, it has been shown that muscle glucose uptake is minimally increased [15], while the time course of plasma insulin and glucose mainly reflects hepatic glucose handling [15], so that the insulin resistance index in the liver can be calculated. We found that a fructose-rich diet elicited an increase in insulin resistance index in the liver (Figure 7B) and decreased activation of insulin downstream effector kinase Akt (Figure 7C). Interestingly, these alterations persisted three weeks after returning to control diet, as indicated by the higher insulin resistance index and lower pAkt/Akt ratio found in FR rats, compared to CR rats (Figure 7A,B). Finally, a lower sensitivity to insulin was also evident in CR rats, compared to C rats, thus evidencing an age-related effect.

**Figure 8:**
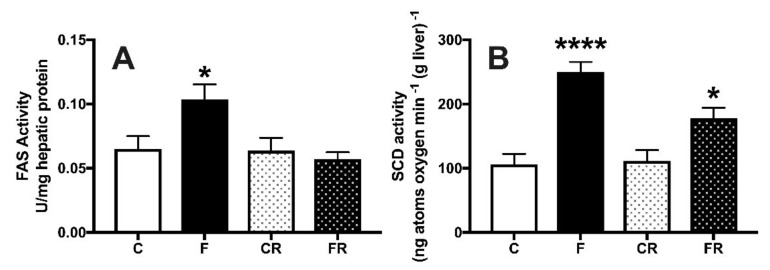
Hepatic activity of fatty acid synthase (**A**) and stearoyl-CoA desaturase (**B**) in control (C), fructose-fed (F), control rescue (CR) and fructose rescue (FR) rats. Values are the means ± SEM of eight different rats. * *p* < 0.05, **** *p* < 0.0001 compared to respective control (one-way ANOVA followed by Tukey post-test).

Fructose is long far known to induce de novo lipogenesis in the liver [27] and accordingly we found that the activity of hepatic FAS (Figure 8A) and SCD (Figure 8B) was significantly upregulated in F rats. Following three weeks of returning to control diet in FR rats, FAS activity returned to control levels (Figure 8A), while that of SCD remained higher (Figure 8B).

### 3.4. Hepatic Mitochondrial Function

**Figure 9:**
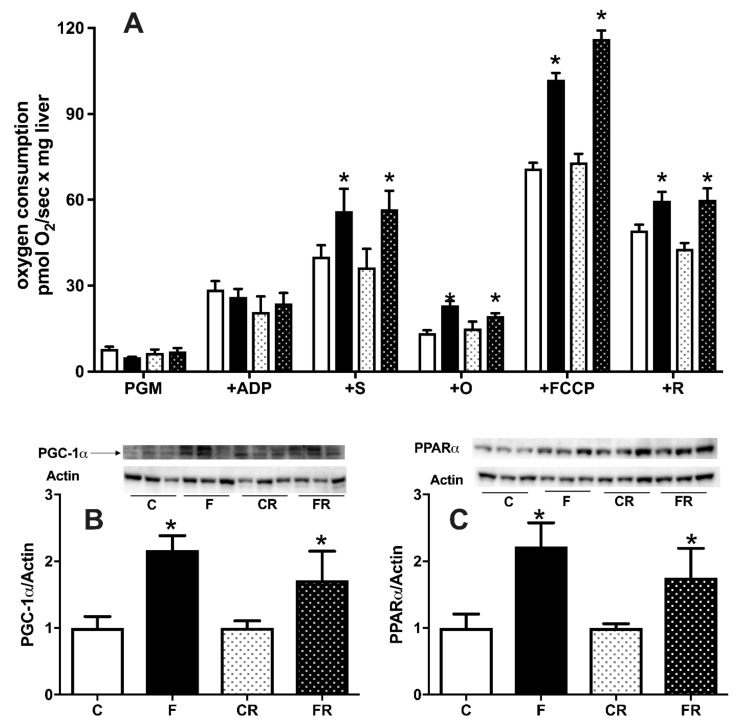
Hepatic mitochondrial respiratory activity (**A**), protein content (with representative western blots) of peroxisome proliferator-activated receptor-gamma coactivator 1 alpha (PGC1α) (**B**), peroxisome proliferator activated receptor alpha (PPARα) (**C**) in control (C), fructose (F), control rescue (CR), and fructose rescue (FR) rats. Mitochondrial respiration (panel A) was measured in the presence of complex I-linked substrates pyruvate + glutamate + malate (PGM), adenosyntriphosphate (ATP), complex II-linked substrate succinate (S), ATP synthase inhibitor oligomycin (O), uncoupler carbonylcyanide p-trifluoromethoxyphenyl-hydrazone (FCCP) and inhibitor of complex I rotenone (R). Values are the means ± SEM of eight different rats. * *p* < 0.05 compared to respective control (one-way ANOVA followed by Tukey post-test).

Given the central role of these organelles for cellular energy handling, we also investigated the function of hepatic mitochondrial compartment. As shown in Figure 9A, three weeks of high fructose intake were able to induce significant changes in hepatic mitochondrial physiology. In fact, respiration driven by complex I and II was significantly upregulated in F rats, both in the presence of ATP and after the inhibition of ATP synthase by oligomycin, while respiration driven solely by complex I was not affected by fructose intake. In addition, when maximal capacity of respiratory chain was measured in the presence of the uncoupler FCCP, a significant increase was evident, even after the addition of rotenone, an inhibitor of complex I. All the above increases were found to persist also three weeks after switching to the control diet. To gain further insight into the regulators of hepatic metabolism, we also assessed the protein amount of two master regulators, PGC1α (Figure 9B) and PPARα (Figure 9C). Both PGC1α and PPARα protein levels were upregulated by three weeks of fructose-rich diet in F rats, and these variations persisted in FR rats, three weeks after switching to the control diet.

## 4. Discussion

Liver physiology is highly affected by increased dietary intake of fructose [11,12], even after short-term treatments [14]. However, less is known about whether these detrimental effects can be reversed, or they rather persist after fructose withdrawn from the diet. To clarify this issue, we first evaluated the effects of three weeks of fructose-rich diet on several metabolic and hepatic parameters and, more importantly, investigated whether the fructose-induced metabolic disorders are reversible after switching to a control diet.

The analysis of the early effect of increased fructose intake on whole body energy balance showed no significant changes in body composition, in agreement with previous results obtained after 2 weeks of fructose-rich diet [14]. Surprisingly, weight gain and lipid gain, as well as the amount of epididymal and retroperitoneal adipose tissue were all found increased after switching to control diet. The expansion of the body lipid compartment was at the expense of accretion of body protein mass. The analysis of macronutrient partitioning revealed that, even three weeks after the cessation of fructose-rich diet, almost all the introduced lipids were deposited in FR rats, while—in CR rats—half of the lipid intake was diverted towards lipid gain, and the other half is oxidized for fueling energetic needs. As a consequence, in FR rats, energy expenditure was maintained at the expense of protein deposition, and this shift in macronutrients partitioning elicited the increased lipid mass typical of obese state, coupled with decreased protein mass. To our knowledge, this is the first report dealing with the recovery of rat’s whole body energy balance from a period of high fructose intake. It has been reported that switching from a high fat to a control diet rapidly restores fat mass in mice [28], thus indicating that the metabolic adaptations to increased fructose intake, and the related health risks, are more prolonged than those caused by increased fat intake. In addition, the young age of the rats poses a further concern on the harmful effect of high fructose intake. In fact, the translation of our results on rodent model to humans implies that the elevated consumption of fructose in young people, by impacting on accretion of lean mass, could exert harmful effects later in adulthood and in middle age. Interestingly, it has been previously found that consuming fructose during forced suckling results in long-lasting increase in body weight and insulin secretion [29]. An intriguing observation comes if we consider that low energy intake in critical periods of development, such as growing phase, is associated with the phenomenon of ‘catch up fat’, in which—at a similar level of caloric intake—rats that have been previously subjected to caloric restriction gained more fat than the control ones [30]. Our present results seem to indicate that fructose consumption during the growing phase elicits a ‘catch up fat’-like response similar to the one induced by caloric restriction, even though during fructose dietary treatment the caloric intake was the same. In addition, hormonal status may be modified by fructose intake and may in turn exert its influence on subsequent weight gain of FR rats during the rescue period.

Recent findings have outlined the sequence of events during high intakes of fructose [9]. When gut metabolism of fructose becomes saturated by excessive dietary intake, a consequent overflow of this sugar in the portal circulation occurs and hence the liver is challenged with high fructose concentrations. The present finding of increased systemic fructose concentrations suggests that also hepatic metabolic capacity is saturated by fructose, while increased plasma uric acid and ALT are indicative of liver damage consequent to massive utilization of this sugar. Plasma inflammatory markers correlate with the above picture, showing a significant increase in LPS, TNFα, haptoglobin, and lipocalin. Most of the above markers were restored to control values after switching to a control diet, with the notable exception of LPS and TNFα, both of which were still higher even three weeks after the cessation of the fructose-rich diet. Since LPS is considered a marker of altered gut permeability, able to induce systemic inflammation, its higher plasma levels in FR rats strongly suggest prolonged gut dysfunction.

In line with previous results obtained in young rats after two weeks of increased fructose intake [14], we found a lower activation of the insulin downstream effector Akt in liver from fructose-fed rats. Interestingly, the lower response to insulin in the liver persisted also in FR rats, in agreement with a very recent finding obtained in mice, showing abnormal glucose tolerance even 24 weeks after switching to control diet [31]. It should be noted that our data on hepatic insulin action are also indicative of reduced action of insulin in CR rats, that are older than C rats. This result can be explained taking into account that transient increase in insulin resistance is a normal component of pubertal development [32,33]. In addition, it is well known that age related insulin resistance appears early during development [34] and hepatic glucose production and glucose-6-phosphatase activity were found decreased 18% and 30%, respectively, between two and four months of age [35].

Of note, two factors known to induce an impairment in the signaling of insulin, i.e., hepatic triglycerides and ceramide [36], returned to control levels after switching to a control diet. Thus, it appears that the reduced functioning of the insulin signaling pathway in the liver is driven by other mechanisms. In this context, a role could be certainly played by LPS and TNFα, since both mediators are able to interfere with insulin signaling [37,38]. We have previously found that long-term fructose-induced hepatic insulin resistance was reversed in concomitance with normalization of plasma levels of LPS and TNFα [12,39]. Therefore, it can be hypothesized that in the present study the persistence of increased levels of LPS and TNFα could participate in the maintenance of insulin resistance in the liver of FR rats. Similarly, the prolonged increase of plasma ALT might not merely depend on steatosis but might also rely on high levels of inflammatory molecules TNF-α and LPS, whose effect on liver injury is reported [40].

In the present study, fructose-induced increase in hepatic content of triglycerides and ceramide is abolished by switching back to control diet. However, an intriguing result is the persistence of elevated plasma levels of triglycerides in fructose-rescued rats. The increase in plasma triglycerides elicited by a fructose-rich diet is due to the stimulation of hepatic de novo lipogenesis and export of neosynthesized lipids into the bloodstream [41]. In agreement, increased activity of FAS and SCD was found in the liver of fructose-fed rats. After switching to a control diet, FAS activity returned to control values, while SCD activity remained significantly higher. Interestingly, it has been found that hepatic levels of SCD mRNA were still higher 14 h after fructose removal from the diet, although the expression of other genes fell to normal levels [42]. It has been demonstrated that SCD activity is positively correlated with export of lipids from the liver and negatively correlated with hepatic steatosis [43]. Thus, it can be hypothesized that the prolonged increase in SCD activity helps to export into the bloodstream the triglycerides that have been deposited in the liver. This mechanism could contribute to the normalization of hepatic triglycerides, as well as to the persistence of elevated plasma triglycerides, as also hypothesized by Kim et al. [44] following nine weeks of a fructose-rich diet.

The enhanced flux of fructose coming from the diet is known to impact on the handling of energy in the liver, since the uncontrolled metabolism of fructose leads to depletion of cellular ATP [45]. A major role in cellular ATP homeostasis is played by the mitochondria, whose function is modified by dietary stimuli [13], so another goal of this study was to clarify their adaptation to fructose overload. Functional analysis of liver mitochondria revealed that fructose-rich diet elicited a stimulation of mitochondrial respiration. In detail, the increased mitochondrial respiration was evident only in the presence of both complex I- and II-linked substrates, but not with only complex I-linked substrates. From these results, it can be inferred that the stimulation of mitochondrial respiration is the result of changes in the steps beyond complex I, i.e., the respiratory chain from complex II onwards, ATP synthase and ATP/ADP carrier. The increased mitochondrial respiration persisted also when maximal capacity of respiratory chain was measured in the presence of the uncoupler FCCP, thus suggesting that ATP synthase and ATP/ADP carrier are not involved in the regulation of mitochondrial respiration. More probably, the point of regulation is located in the respiratory chain from complex II onwards, as also indicated by the fact that the increased respiration persisted even after the addition of rotenone, an inhibitor of complex I. Interestingly, in a previous study it has been found increased mitochondrial gene expression and mtDNA content in the liver from young rats receiving a 20% fructose solution for 14 weeks [46]. The increased mitochondrial respiratory capacity could be envisaged as a feedback response that tries to compensate the ATP depletion generated by fructose metabolism. The increased mitochondrial oxidative capacity well correlates with increased protein content of PGC-1α, which is known to control the respiratory capacity of mitochondria [47], as well as the increased protein content of PPARα, a known transcription factor induced by increased fatty acid supply to liver cells. In line with our data, PGC-1α has been found increased in the liver of diabetic mouse models [48], and it has been shown to inhibit insulin signaling in the liver [49].

In conclusion, we show prolonged fructose-induced dysregulation of the hepatic metabolism, even after switching to a control diet. This finding demonstrates the risk of an unhealthy sugar-rich diet that can have long lasting consequences despite the return to a healthy diet. This unexpected finding could have implications for future treatment regimens of liver disease in humans.

## Figures and Tables

**Table 1:** Ingredients and nutritional composition of experimental diets.

Ingredients, g/100 g	Control Diet	Fructose Diet
Standard chow ^a^	50.5	50.5
Sunflower oil	1.5	1.5
Casein	9.2	9.2
Alphacel	9.8	9.8
Cornstarch	20.4	---
Fructose	---	20.4
Water	6.4	6.4
AIN-76 mineral mix	1.6	1.6
AIN-76 vitamin mix	0.4	0.4
Choline	0.1	0.1
Methionine	0.1	0.1
**Energy content and composition**		
Gross energy density (kJ/g)	17.2	17.2
ME content (kJ/g) ^b^	11.1	11.1
Proteins (% ME)	29.0	29.0
Lipids (% ME)	10.6	10.6
Carbohydrates (% ME)	60.4	60.4
Of which:		
Fructose	---	30.0
Starch	52.8	22.8
Sugars	7.6	7.6

^a^ 4RF21, Mucedola, Italy; ^b^ Estimated by computation using values (kJ/g) for energy content as follows: proteins 16.736, lipids 37.656, and carbohydrates 16.736. ME = metabolizable energy.

**Table 2:** Body weight and energy balance after three weeks of high fructose feeding or after three weeks of control diet following three weeks of high fructose feeding.

Variables	C	F	CR	FR
Initial body weight, g	170 ± 5	170 ± 5	310 ± 4	306 ± 4
Final body weight, g	310 ± 3	306 ± 5	370 ± 6	388 ± 3 ****
Body weight gain, g	140 ± 4	136 ± 3	60 ± 4	82 ± 4 **
Epididymal pad weight, g	2.8 ± 0.1	2.8 ± 0.1	3.7 ± 0.1	4.8 ± 0.1 *
Epididymal pad weight, g/100 g b.w.	0.91 ± 0.05	0.92 ± 0.05	1.01 ± 0.05	1.31 ± 0.06 ***
Retroperitoneal pad weight, g	1.8 ± 0.1	1.7 ± 0.1	3.1 ± 0.1	4.4 ± 0.2 *
Retroperitoneal pad weight, g/100 g b.w.	0.61 ± 0.04	0.62 ± 0.03	0.84 ± 0.04	1.13 ± 0.05 ***
ME intake, kJ	8979 ± 203	9039 ± 201	9007 ± 98	9107 ± 98
Energy gain, kJ	1227 ± 15	1112 ± 59	700 ± 46	1035 ± 50 ****
Energy expenditure, kJ	7851 ± 217	8442 ± 173	8335 ± 229	8071 ± 308
Energetic efficiency, %	13.6 ± 0.3	11.7 ± 0.7	7.5 ± 0.5	8.2 ± 0.9

Values are the means ± SEM of eight different rats. * *p* < 0.05, ** *p* < 0.01, *** *p* < 0.001, **** *p* < 0.0001 compared to respective control (one-way ANOVA followed by Tukey post-test). ME= metabolizable energy; control (C), fructose (F), control rescue (CR), fructose rescue (FR). Energy balance values are referred to three weeks of control diet (C) or fructose-rich diet (F), or three weeks of control diet given to rats following the period of control diet feeding (CR) or fructose-rich diet feeding (FR).

## Data Availability

Data are contained within the article.
